# Renin–Angiotensin Inhibitor, Captopril, Attenuates Growth of Patient-Derived Colorectal Liver Metastasis Organoids

**DOI:** 10.3390/ijms25063282

**Published:** 2024-03-14

**Authors:** Georgina E. Riddiough, Theodora Fifis, Vijayaragavan Muralidharan, Christopher Christophi, Bang M. Tran, Marcos V. Perini, Elizabeth Vincan

**Affiliations:** 1 Department of Surgery, Austin Health Precinct, The University of Melbourne, Austin Health, Lance Townsend Building, Level 8, 145 Studley Road, Heidelberg, VIC 3084, Australia; georgina.riddiough@unimelb.edu.au (G.E.R.); tfifis@unimelb.edu.au (T.F.); v.muralidharan@unimelb.edu.au (V.M.); c.christophi@unimelb.edu.au (C.C.); 2 Department of Infectious Diseases, The University of Melbourne, The Peter Doherty Institute, Melbourne, VIC 3000, Australia; manht@unimelb.edu.au; 3 Victorian Infectious Diseases Reference Laboratory, The Peter Doherty Institute, Melbourne, VIC 3000, Australia; 4 Curtin Medical School, Curtin University, Perth, WA 6102, Australia

**Keywords:** colorectal liver metastasis, renin–angiotensin inhibitors, organoids

## Abstract

The recurrence of colorectal liver metastasis (CRLM) following liver resection is common; approximately 40% of patients will experience tumor recurrence post-surgery. Renin–angiotensin inhibitors (RASis) have been shown to attenuate the growth and progression of CRLM in pre-clinical models following liver resection. This study examined the efficacy of the RASi captopril on patient-derived colorectal liver metastasis organoids. Patient-derived organoids (PDOs) were established using fresh samples of colorectal liver metastasis from appropriately consented patients undergoing liver resection. To mimic the regenerating liver post-CRLM liver resection, PDOs were cultured under hepatocyte regeneration conditions in vitro. CRLM PDOs were established from three patients’ parent tissue. CRLM PDOs and parent tissue expressed markers of colorectal cancer, CDX2 and CK20, consistently. Furthermore, CRLM PDOs treated with captopril showed a dose dependent reduction in their expansion in vitro. In conclusion, CRLM PDOs recapitulate in vivo disease and displayed a dose-dependent response to treatment with captopril. RASis may be an additional viable treatment for patients with CRLM.

## 1. Introduction

Renin–angiotensin system inhibitors (RASis) are commonly prescribed for hypertension and cardiac failure. Clinical and experimental data have shown that RASis may benefit cancer patients. RASis are associated with prolonged survival in rectal cancer patients [1] and reduce recurrence for a variety of solid tumors [2,3,4]. Experimental data have shown that RASis possess a variety of antitumor properties, including immunomodulatory, anti-angiogenic, and anti-proliferative effects [5,6,7,8]. Previously, we demonstrated in vivo that treatment with captopril, a RASi, attenuates the growth of colorectal cancer liver metastases (CRLM) in the regenerating liver following partial hepatectomy [9]. We also showed that this was associated with changes in the tumor immune microenvironment [9] and tumor-specific downregulation of Wnt target genes’, *c-myc* and *cyclin D1*, expression [10]. The objectives of this study were to establish and characterize patient-derived CRLM organoids and build on our prior findings by assessing the efficacy of captopril on human CRLM organoid growth under liver regeneration conditions in vitro.

Organoids are mini-replicas of tissues grown in a dish and recapitulate the key characteristics and function of their tissue of origin. They are self-organizing three-dimensional structures that can be established either from pluripotent stem cells (such as embryonic stem cells or induced pluripotent stem cells) or adult stem cells isolated from mature organs [11]. Organoids can also be established from patient tumors and are referred to as patient-derived organoids (PDOs). PDOs have recently become a popular platform for oncological therapeutic assessment as they offer several advantages over conventional cell lines and other experimental models, such as human cancer cell line xenografts and patient tumor-derived xenografts (PDX) [12]. PDOs retain the genetic and phenotypic features of their parent tumor in long-term passage and can be cryopreserved, facilitating the establishment of human cancer biobanks [13,14]. Additionally, unlike PDX animal models, PDOs are tissue culture-based and facilitate ready access to genetic and pharmacological manipulation as well as advanced imaging techniques. One of the most exciting applications of PDOs has been drug screening, which has informed alterations to patient therapy in real time [15].

Wnt/β-catenin signaling and organoid establishment are inextricably linked. The establishment of epithelial organoids derived from adult stem cells relies on the isolation and culture of Lgr5+ stem cells, Lgr5 being a key Wnt target gene. These cells, when grown within a basement membrane matrix in serum-free conditions, which mimic the stem cell niche, form 3D structures which closely resemble the parent tissue. One major component of the stem cell niche and organoid cell culture medium is R-spondin, a Wnt potentiator and Lgr5 ligand [16,17]. As well as being a marker of adult stem cell populations within the intestinal epithelium [18], liver [19], stomach [20], and ovary [21], amongst others, Lgr5 is also a marker of colorectal cancer (CRC) stem cells [22,23,24]. Wnt signaling is also central to CRC carcinogenesis. The majority (>80%) of CRCs will harbor gene mutations in the Wnt signaling pathway, most commonly involving the truncation of the *Adenopolyposis Coli* (*APC*) gene, but other mutations within the pathway can also drive aberrant Wnt signaling [25,26]. These mutations culminate in constitutive activation of Wnt signaling, which is evident histologically as the accumulation of cytoplasmic and nuclear β-catenin. This drives the constitutive transcription of Wnt target genes, leading to CRC carcinogenesis [27]. For these reasons, and in view of our prior reports that the RASi captopril induced the tumor-specific downregulation of Wnt target genes [10], we investigated the effect of captopril treatment on the Wnt target gene in CRLM PDOs.

## 2. Results

To mimic CRC metastatic tumor growth following partial hepatectomy, CRLM PDOs were established and cultured in a liver regeneration medium in vitro [28,29]. Nineteen patients undergoing liver resection for CRLM were consented and recruited into this study. Fresh tissue samples were collected from 17 patients, and 13 of these underwent tissue processing for the establishment of CRLM PDOs, as well as “normal” liver organoids from far liver non-cancer tissue. CRLM PDOs were successfully established for three patients (3/13, 23%). This was surprising given that some groups have reported success rates of up to 71% (10/14), with eight of these (57%) suitable for further genomic analysis [30]. On the other hand, “normal” liver organoids were successfully established from all 17 patients. It may seem counterintuitive that non-cancerous liver PDOs are easier to grow than CRLM PDOs, however, challenges with CRC culture have also been experienced by others in the field [31], indicating a lack of laboratory-to-laboratory standardization of protocols.

### 2.1. Patient-Derived Organoids Generated from Three Patients with Colorectal Liver Metastasis

The three PDOs established from three patients undergoing liver resection for CRLM were investigated further. All patients were confirmed as having metastatic CRLM on final histopathology (Table 1). Two patients received neo-adjuvant chemotherapy, relative to their liver resection(s) (Table 1).

### 2.2. Patient #1

Patient #1, a 49-year-old female, was diagnosed with CRC in 2016. Patient #1 has no significant past medical history; however, she is an active smoker. Patient #1 completed a course of neoadjuvant chemoradiotherapy (50.4 Gray in 28 fractions with capecitabine) prior to undergoing an ultra-low anterior resection for pT3N1 rectal adenocarcinoma. She also underwent a laparoscopic segment VI resection for a synchronous liver metastasis followed by eight cycles of adjuvant FOLFOX (folinic acid, 5-fluorouracil, and oxaliplatin) with 5-FU (5-fluorouracil) alone from cycle five due to peripheral neuropathy. Subsequent surveillance scanning detected two metachronous segment VIII colorectal liver metastases. She underwent segment VIII liver resection in May 2019 and did not receive additional chemotherapy prior to this. Rectal tissue histopathology and genetic analysis revealed this patient to have a proficient mismatch repair (pMMR) CRC with wild-type KRAS and BRAF expression (Table 1). The histopathology of the resected liver lesions confirmed they were CRLM and the background liver was noted to be approximately 30% macrosteatotic. At the last follow-up, 24 months post-second liver resection, this patient was found to have a new liver lesion which was recently treated with microwave ablation.

**Figure 1:**
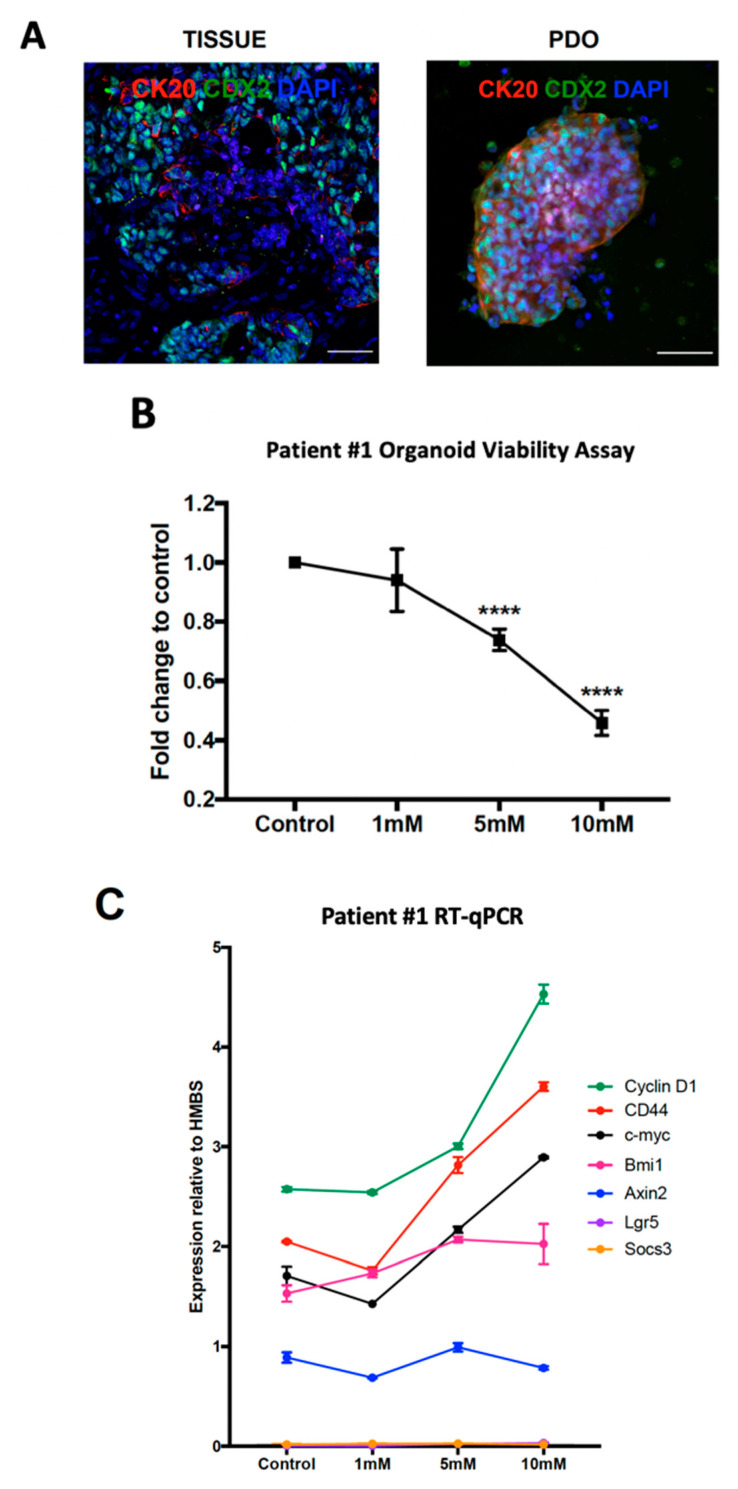
Patient #1 Organoids. Patient-derived organoids (PDOs) were established from the tumor of Patient #1 who underwent liver resection for colorectal liver metastasis (CRLM). Fresh tissue specimens were obtained and used to establish organoid cultures and a portion of parent tissue was formalin fixed. (**A**) Immunofluorescence (IF) characterization of CRLM PDOs with markers to confirm colorectal cancer, CDX2 (green) and CK20 (red) (scale bar is 50 μm). Parent tumor tissue and PDO expression of CDX2 and CK20 are consistent. (**B**) Patient #1 PDOs were treated with increasing concentrations of captopril and CRLM PDO growth assessed using the MTT assay (mean ± SEM, *n* = three biological repeats were performed [three separate organoid preparations] and eight technical replicates of each assessed). Statistical significance was determined using the Student’s *t*-test. **** *p* < 0.0001. (**C**) Quantitative reverse transcriptase (RT-qPCR) analysis of Patient #1 PDOs following treatment with increasing concentrations of captopril (mean ± SEM, *n* = 3 technical repeats). Gene expression was determined relative to housekeeping gene, *HMBS*.

The immunofluorescence (IF) characterization of both the parent tumor tissue and PDOs was undertaken to establish whether the expression of IF markers was consistent in both the parent tissue and the PDO cultures. CDX2 and CK20 staining is routinely undertaken by pathologists to confirm that metastatic lesions are CRC. Parent tumor tissue and PDOs both demonstrated nuclear CDX2 staining, alongside membranous CK20 staining (Figure 1A, Figures S1 and S2). These data confirm that Patient #1 PDOs recapitulate the histology of the parent tissue. Patient #1 PDOs responded in a dose-dependent manner to treatment with captopril (Figure 1B).

Next, we assessed the expression of various Wnt target genes including *c-myc* [32], *cyclin D1* [33,34], *Axin2* [35], *CD44* [36], *Bmi1* [37], *Lgr5* [14], and *Socs3* gene expression. *Socs3* is a gp130-Jak-Stat signaling pathway target gene. The Wnt/β-catenin and gp-130-Jak-stat pathways cross-communicate and it has been shown that the inhibition of the gp-130-Jak-stat3 pathway slows the growth of *APC* mutated CRCs [38]. Patient #1 PDOs expressed high levels of the Wnt target genes *cyclin D1*, *CD44*, *c-myc*, and *Bmi-1* following treatment with captopril (Figure 1C), which is in stark contrast to our previously published in vivo murine findings. However, the expression of other Wnt target genes including *Axin2*, commonly referred to as the universal Wnt target gene, and *Lgr5* did not alter with captopril treatment. Additionally, the expression of *Socs3* did not change.

### 2.3. Patient #2

Patient #2, a 75-year-old female, was diagnosed with CRC in 2018. Patient #2 has a past medical history of gastro-esophageal reflux disease (GORD) and is a non-smoker. She underwent a right hemicolectomy for pT2N1 colorectal adenocarcinoma and completed eight cycles of adjuvant capecitabine. Routine surveillance scanning detected a segment VIII CRLM and subsequent magnetic-resonance imaging of the liver revealed three suspicious lesions all within the right side of the liver. Following a multi-disciplinary meeting (MDM), it was recommended that the patient undergo neoadjuvant chemotherapy prior to liver resection. Patient #2 completed six cycles of FOLFOX and then underwent multiple subsegmental liver resections in December 2019. Colorectal tissue histopathology and genetic analysis revealed that this patient had a pMMR CRC with wild-type KRAS expression. BRAF mutation assays were not performed for this patient (Table 1). The histopathology of the resected liver lesions confirmed they were CRLM and showed the background liver to be healthy with very minimal (1%) macrosteatosis. At the last follow-up, 18 months following liver resection, this patient was noted to have a new liver lesion suspicious for disease recurrence, but has declined ongoing investigation and treatment.

**Figure 2:**
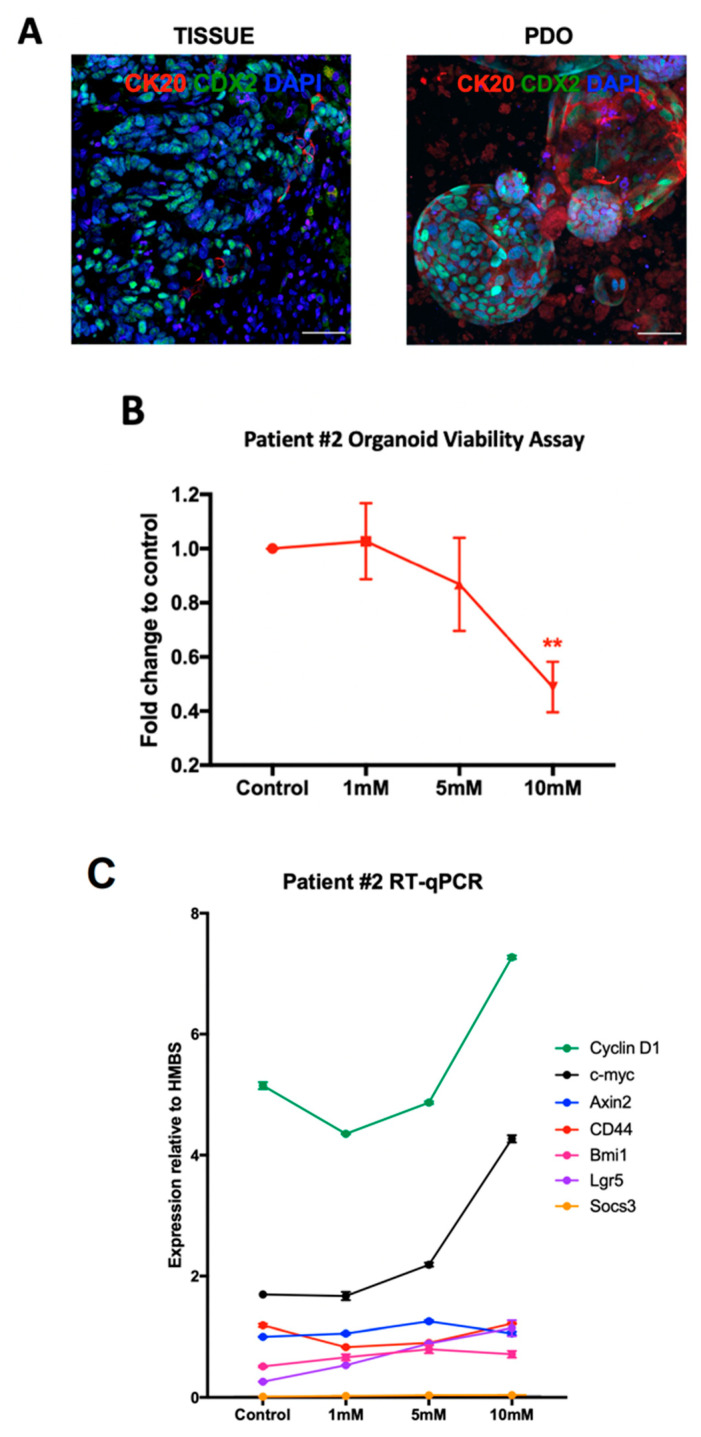
Patient #2 Organoids. Patient-derived organoids (PDOs) were established from the tumor of Patient #2 who underwent liver resection for colorectal liver metastasis (CRLM). Fresh tissue specimens were obtained and used to establish organoid cultures and a portion of parent tissue was formalin fixed. (**A**) Immunofluorescence (IF) characterization of CRLM PDOs with markers to confirm colorectal cancer, CDX2 (green) and CK20 (red) (scale bar is 50 μm). Parent tumor tissue and PDO expression of CDX2 and CK20 are consistent. (**B**) Patient #2 PDOs were treated with increasing concentrations of captopril and CRLM PDO growth was assessed using the MTT assay (mean ± SEM, *n* = three biological repeats were performed [three separate organoid preparations] and eight technical replicates of each assessed). Statistical significance was determined using the Student’s *t*-test. ** *p* < 0.01. (**C**) Quantitative reverse transcriptase (RT-qPCR) analysis of Patient #2 PDOs following treatment with increasing concentrations of captopril (mean ± SEM, *n* = three technical repeats). Gene expression was determined relative to housekeeping gene, *HMBS*.

The IF characterization of the PDOs confirmed that the PDO expression of CDX2 and CK20 matched that of the fixed parent tumor tissue (Figure 2A). Patient #2 PDOs were treated with captopril in vitro and also demonstrated a dose-dependent response (Figure 2B). Patient #2 PDOs exhibited a greater expression of certain Wnt target genes with increasing captopril treatment. The expression of *cyclin D1*, *c-myc*, *Bmi-1*, and *Lgr5* all increased in response to increasing concentrations of captopril treatment (Figure 2C). The expression of the gp-130-Jak-Stat3 signaling target gene *Socs3* did not alter with captopril treatment (Figure 2C).

### 2.4. Patient #3

Patient #3, a 58-year-old male, was diagnosed with CRC and synchronous CRLM in 2020. Patient #3 has a past medical history of GORD and chronic back pain. He is a non-smoker. Patient #3 completed 12 cycles of neoadjuvant FOLFIRI (folinic acid, 5-fluorouracil, and irinotecan) prior to an anterior resection for a pT4N1 CRC. Colorectal histopathology and genetic analysis revealed this patient had a pMMR CRC with wild type BRAF, but mutant KRAS expression (Table 1). The patient underwent an ALPPS (associating liver partition and portal vein ligation for staged hepatectomy) procedure in May 2020, the second stage of which was completed three weeks later. Histopathology confirmed the resected liver lesions were CRLM and demonstrated that the background liver was non-cirrhotic with approximately 10% macrosteatosis. At the last follow-up, eleven months following liver resection, this patient was found to have progressed on systemic treatment and was transitioned to the best supportive care.

PDOs were established from fresh CRLM tissue obtained from this patient at the time of the first stage of the ALPPS. Patient #3’s PDOs grew very slowly and could only be passaged up to eight times. Consequently, less organoid material could be generated for experimental purposes. Enough material was obtained to perform the organoid viability assays to assess the efficacy of captopril on Patient #3’s PDO growth in vitro. However, not enough material could be generated to include the IF characterization or RT-qPCR analysis. RT-qPCR analysis was undertaken despite the small amount of organoid material obtained, but this was insufficient to detect all genes. Results could be obtained for *Axin2* and *Socs3*, but not for the housekeeping gene *HMBS*, which meant that we could not correct the quality of the RNA between samples. However, the raw data demonstrated that *Axin2* expression decreased and *Socs3* expression increased slightly (1 Ct cycle) with captopril treatment, which may simply reflect the quality of the RNA, as these genes did not change for the Patient #1 and #2 samples (Table S1). The organoid viability assay on Patient #3 demonstrated that this patient’s CRLM PDOs also responded to treatment with captopril (Figure S3), although the response was less dramatic than that observed with Patient #1 and #2 (Figure 1 and Figure 2).

## 3. Discussion

This study demonstrates that CRLM PDOs recapitulated in vivo disease histopathology and displayed a dose-dependent response to treatment with captopril. The result translates previous work in mice by our laboratory which demonstrated that the RASi captopril attenuates the progression of CRLM in the regenerating and non-regenerating liver [9,39]. In murine experiments, this finding was accompanied by the downregulation of Wnt target genes, *c-myc* and *cyclin D1* [10]. However, this study demonstrated that captopril treatment was associated with the increased expression of Wnt target genes in PDOs. These findings most likely suggest that the surviving PDOs consist of highly resistant cancer stem cells which express high levels of anti-apoptotic proteins such as *c-myc*, and stem cells markers such as *Lgr5*. Wnt/β-catenin signaling has been implicated in the development of drug resistance and the persistence of cancer stem cells [40,41]. In this study, we did not assess the activity of the Wnt signaling pathway directly, so it remains to be determined whether the persistence of resistant cancer stem cells is due to aberrant Wnt signaling or not. However, despite captopril successfully attenuating CRLM PDO growth in vitro, the persistence of populations of cancer stem cells is clinically important. These surviving cancer stem cells may lead to disease recurrence and future studies will examine the potential of these cells to re-establish metastatic disease and, moreover, develop drug therapies against this population of residual cancer stem cells to combat disease recurrence. Additionally, these findings highlight the importance of developing combination therapies for cancer to achieve long-lasting remission and circumvent drug resistance.

The discordance between our in vivo murine studies and this human PDO study also highlights an important limitation of current organoid technology, most likely due to the lack of representation of the in vivo tumor microenvironment (TME). In this study, we did not recapitulate other components of the TME alongside PDOs. Whilst PDOs have been shown here and by others to successfully recapitulate in vivo disease, to date cancer organoid cultures do not have the complexity to mimic the complete TME [12]. A typical tumor normally contains populations of stromal cells, fibroblasts, endothelium, as well as immune cells. Although more complex organoid systems are being explored, such as organoid co-cultures with immune [42,43] and endothelial [44] cells, none have established a complex organoid system that successfully incorporates all the cell types observed within the TME in vivo.

Studies have shown retrospectively that PDOs reliably display the same responses to chemotherapy in vitro as matched patients displayed in vivo [45,46]. Other studies have gone further and demonstrated that organoids established prospectively can inform treatment decisions for patients [15]. Although we did not set out to replicate the responses of chemotherapy in vitro, these studies highlight that PDOs do respond in a way which mimics the patient’s clinical response. These results suggest that where we observed a beneficial response to treatment with captopril in vitro, this could be expected in vivo. However, a clinical trial comparing the outcomes of patients and their PDOs in response to captopril treatment is required to investigate this further.

One limitation of this study is the poor success rate of establishing CRLM PDO cultures. The competition between the CRLM tumor and normal liver cells in the initial phase of establishing CRLM PDOs under liver-regenerating conditions is one possible contributing factor. The outgrowth of the normal liver cells present in the resected tumor tissue required the careful teasing apart of CRLM PDOs from normal liver PDOs. Patient and CRLM intrinsic factors may also dictate PDO growth. Patient #1 grew the most easily expandable PDOs and this could be because this patient did not receive chemotherapy prior to liver resection. This is a difficult problem to overcome since most patients undergoing liver resection for CRLM will receive neoadjuvant chemotherapy. Clinical evidence supports the administration of neoadjuvant chemotherapy for these patients because it has been shown to prolong overall survival [47]. Nevertheless, this would be the case for other centers also culturing CRLM PDOs who have reported greater success rates. Notably, however, Patient #2, whose organoids also grew well, did receive neoadjuvant chemotherapy with six cycles of FOLFOX. The histopathology of the resected liver specimens demonstrated that the background liver was healthy with no signs of cirrhosis or chemotherapy-associated steatohepatitis. This implies that although this patient received neoadjuvant therapy, there was no adverse effect observed in the hepatocytes and this may explain why PDOs were relatively easy to establish for this patient. Patient #3 PDOs were persistently very slow growing. Interestingly, Patient #3 was the only patient included in this study whose CRC was associated with a Kras mutation. In health, upstream EGFR activation stimulates Kras. When mutated, Kras signaling is constitutively switched on and leads to increased signaling in various pathways such as mTOR, PI3K, AKT, and ERK. In the clinic, Kras mutations identify patients that may respond to anti-EGFR therapies such as cetuximab and bevacizumab. The presence of Kras mutations could be assumed to enhance PDO growth by activating cell survival and proliferation pathways; however, we found that Patient #3 PDOs were more difficult to grow. The PDO media utilized in this study contain human EGF, and it is therefore possible that excessive EGFR signaling due to the combination of a Kras mutation and additional EGFR activation by EGF in the media leads to cancer cell apoptosis.

Additionally, Patient #3 received twice as many cycles of neoadjuvant chemotherapy as Patient #2. Furthermore, Patient #3 received FOLFIRI instead of FOLFOX. Despite the clinical dogma that different chemotherapeutic regimens cause different degrees of liver toxicity, this has not been definitively borne out by clinical studies [48]. In the future, to try to obtain a greater overall success rate for establishing CRLM PDOs, different media should be trialed taking into consideration the genetic profile of the patient. For Kras mutant tumors, it may be worth investigating whether EGF-free medium conditions improve PDO growth. Recently, it has also been shown that the sequential knockout of the four most commonly mutated genes in CRC, *APC*, *p53*, *Kras*, and *Smad4*, in intestinal organoids leads to the development of invasive carcinomas when injected subcutaneously into nude mice [49]. Furthermore, these quadruple knock-out intestinal organoids could be successfully maintained in vitro in the absence of the stem cell niche factors commonly used to supplement patient-derived organoid medium, including R-spondin [49,50]. This also raises the possibility that the addition of R-spondin to the medium used in our study to grow CRLM PDOs may not be necessary. R-spondin potentiates Wnt signaling [51], however in *APC*-mutated CRCs, Wnt signaling is already constitutively switched on [27]. Since the majority of CRCs harbor a mutation of *APC*, or another mutation within the Wnt/β-catenin pathway [25], the exclusion of the Wnt signaling potentiator, R-spondin, and other stem cell niche factors from CRLM PDO culture medium should also be investigated in future studies.

In the future, the response of CRLM PDOs to in vitro radiotherapy could also be assessed. Radiotherapy, in the form of either selective internal radiation therapy (SIRT) or stereotactic body radiotherapy (SBRT), is non-surgical, and usually there are palliative treatment options for patients with CRLM and other hepatic malignancies [52,53]. Patients #1 and #2 developed intrahepatic recurrence following liver resection despite demonstrating different expression patterns for the stem cell marker *Lgr5*. However, distinct gene expression signatures could inform decisions about the use of additional treatments such as radiotherapy. Patient #2’s CRLM PDOs demonstrated high levels of the stem cell marker *Lgr5* following treatment with captopril and this may indicate resistance to radiotherapy. Studies have shown that although Lgr5+ stem cells in the intestine appear highly sensitive to radiation therapy, those in the colon are not [54,55]. This could inform treatment decisions away from therapies such as SIRT or SBRT and towards other therapies, such as microwave ablation, targeted chemotherapies, or monoclonal antibodies. PDOs have the potential to harness personalized medicine, not only by informing treatment decisions regarding chemotherapeutics, but other treatment modalities including radiotherapy.

## 4. Materials and Methods

### 4.1. Human Ethics

This study was conducted in accordance with the Declaration of Helsinki and was approved by the Human Research Ethics Committee (HREC/14/Austin/388) at Austin Health. Patients were recruited between 2018 and 2021 by a surgical member of the research team and provided written consent. Fresh tissue samples were obtained from consenting adults undergoing liver resection for colorectal liver metastasis at Austin Health. Tissue samples were utilized for organoid generation and quantitative reverse transcriptase polymerase chain reaction (RT-qPCR) analysis, and were formalin-fixed for immunohistochemistry.

### 4.2. Patient Data

The patient data collected included patient demographics, the treatment regime, the pathological staging of the primary CRC, and the final histopathology report pertaining to the secondary colorectal liver metastases, size, and number of liver metastases and patient survival.

### 4.3. Establishing CRLM Organoids

The fresh tissue samples of CRLM and corresponding ‘distant liver’ tissue specimens were obtained from consenting patients. The ‘distant liver’ specimens were obtained from a point as far from the tumor as possible and were processed for establishing “normal” liver organoids as previously described [28]. “Normal” refers to the establishment of organoids from adult liver stem cells derived from ‘distant liver’ non-tumoral liver parenchyma, whilst accepting that these patients may have underlying steatosis or sinusoidal injury at baseline or related to the chemotherapy they received. In human patients, the obtainment of ‘distant liver’ tissue is the closest to healthy tissue we can obtain.

Tumor specimens were processed for establishing CRLM organoids under the liver-regenerating conditions previously described [28]. In brief, tissue samples were weighed, dissected, and minced finely into small pieces using a sterile scalpel and scissors. Samples were placed in 15 mL tubes and washed twice with cold DMEM to remove erythrocytes and fat. The samples were allowed to settle in cold DMEM on ice. After that, 12–13 mL of the supernatant was aspirated. For each gram of tissue, 5 mL of pre-warmed liver digestion solution (DMEM supplemented with 2.5 mg/mL of Collagenase D and 0.1 mg/mL of DNase I) was added to the sample. The digestion process was carried out in a 37 °C water bath for at least 30 min with frequent inverting. When the suspension contained 80–100% single cells, digestion was stopped by adding cold DMEM to fill the 15 mL tube. The digested materials were then filtered (40 μm cell strainers) and the filtrate was collected in 50 mL tubes. The tubes were topped up with cold DMEM. The pellets were collected by centrifuging at 400 *g* for 5 min at 4 °C, followed by transferring to 15 mL tubes for repeated washing with cold DMEM. After the last wash, the supernatants were discarded, and for each 1000 cells, 50 μL of Cultrex Reduced Growth Factor Basement Membrane Extract Type 2 (BME2) matrix (R&D Systems, #3533-010-02, Minneapolis, MN, USA) was used to resuspend the pellets. The resuspension was seeded as 15 μL droplets on a prewarmed 6-well plate (Corning Costar #3516, Corning, NY, USA), with 10 droplets per well. The plates were then inverted and incubated for 15 min at 37 °C. Then, 2 mL of warm human liver expansion media was added to each well. The human liver expansion media were made up from Advanced DMEM/F12 (Gibco, #12634010, Waltham, MA, USA) supplemented with 1% Glutamine, 1% penicillin/streptomycin (Gibco, #10378016, Waltham, MA, USA), 10 mM of HEPES (Gibco, #15630080, Waltham, MA, USA), 10% (*v*/*v*) R-spondin1 conditioned medium, 10 mM of nicotinamide (Sigma-Aldrich, #N0636, St Louis, MO, USA), 50 ng/mL of human EGF (Peprotech, #AF-100-15, Cranbury, NJ, USA), 1 mM of N-acetylcysteine (Sigma-Aldrich, #A0737-5MG, St Louis, MO, USA), 25 ng/mL of human HGF (Peprotech, #100-39, Cranbury, NJ, USA), 10 nM of gastrin (Sigma-Aldrich, #G9145, St Louis, MO, USA), 100 ng/mL of FGF10 (Peprotech, #100-26, Cranbury, NJ, USA), 10 μM of Forskolin (Tocris Bioscience, #1099, Bristol, UK), and 5 μM of A83-01 (Tocris Bioscience, #2939 Bristol, UK).

### 4.4. Dosing Calculations

Captopril, a renin–angiotensin inhibitor, has been shown to have a variety of antitumor effects. Studies have shown that the inhibition of renin–angiotensin signaling modulates multiple processes involved in tumor biology such as epithelial-to-mesenchymal transition, cell proliferation and migration, angiogenesis, tumor-immune responses, and extra-cellular matrix remodeling [5]. In order to translate prior murine findings into this pre-clinical patient-derived organoid model, dosing calculations were performed to determine an appropriate in vitro dose. For in vivo murine experiments, a dose of 250 mg/kg/day of captopril was used [9]. On average, mice weighed 25–30 g and were given a single dose of approximately 25 mg/mL of captopril daily, via intra-peritoneal injection, and 25 mg/mL is equivalent to 115 mM (the molecular weight of captopril is 217.3 g/mol). Thus, doses between 10 µM and 10 mM were trialed in this study.

### 4.5. Organoid Cell Viability Assay

PDOs were passaged and resuspended in 10 µL of BME2 per well of a flat-bottom 96-well tissue culture plate (Costar, #3599, Corning, NY, USA). In brief, when the organoids were approximately 80% confluent, the BME2 matrix containing the organoids was disrupted by pipetting with cold DMEM. The dispersed organoids were collected into 15 mL tubes. The tubes were topped up with cold DMEM and centrifuged at 400 *g* for 5 min at 4 °C. After discarding the supernatant, the pellets were broken down further by vigorous pipetting using 2 mL of cold DMEM. The tubes were then topped up and centrifuged again at 400 *g* for 5 min at 4 °C. Finally, the supernatant was removed, and pellets were resuspended in an appropriate amount of BME2 matrix and plated accordingly.

Preliminary viability assays were set up using Patient #1’s PDOs, and a wide range of captopril doses, from 10 μM to 10 mM, were assessed to establish the doses of captopril that would be used (Figure S4). Consequently, doses of 1 mM, 5 mM, and 10 mM of captopril were selected for the experiments.

For each experimental condition, 8 replicate wells were seeded. PDOs were cultured for 72 h in total, with either control human liver expansion medium or human liver expansion medium supplemented with 1 mM, 5 mM, or 10 mM of captopril (S-3 mercapto-2-methylpropionyl-L-proline, Sigma-Aldrich #62571-86-2, St Louis, MO, USA). After 24 h, the PDOs were redispersed and resuspended in 10 μL of the BME2 matrix and seeded onto a flat-bottomed 96-well plate. Fresh control or treatment media were applied. At 48 h, the media were aspirated and replaced with a warm control or treatment media as appropriate. After 72 h, the control or treatment media were carefully aspirated and replaced with 100 µL of warm human liver expansion media plus 50 µL of MTT and incubated for 6 h at 37 °C. Following this, the formazan product was dissolved in SDS and the OD was measured at 570 nm wavelength [56]. For each patient, 3 experiments were performed (i.e., 3 biological repeats for each PDO).

### 4.6. Immunofluorescence Confocal Microscopy

Fresh tissue samples of CRLM were formalin-fixed for 24 h and then transferred to 80% alcohol solution. Samples were paraffin-embedded at Austin Health Pathology and slide-mounted (4 µm sections, mounted on SuperFrost slides [Menzel-Glaser]). The tissue sections were de-waxed and antigen retrieval was performed with Tris buffer (DAKO, S2367, Glostrup, Denmark), pH 9 at 99 °C for 30 min. The slides were incubated with 10% normal goat serum in PBS for 40 min and then with primary antibodies for CDX2 (1:250, Dako IR080) and CK20 (1:250, Ventana, SP33, Tucson, AZ, USA), at 4 °C overnight. The primary antibodies were kindly gifted by Austin Health Pathology. The secondary antibodies, goat anti-mouse 488 (Invitrogen, AlexaFluor, 948490, Waltham, MA, USA) 1:1000 and goat anti-rabbit 568 (LifeTechnologies, AlexaFluor, A11036, Waltham, MA, USA) 1:1000, were then applied for 2 h at room temperature. The slides were also incubated with anti-mouse and anti-rabbit control primary antibodies. The slides were then incubated with DAPI, 1:2000, for 15 min in the dark at room temperature for nuclear staining. Fluorescent mounting medium (Merck, FluorSave Reagent, 345789, Darmstadt, Germany) was applied and the slides were preserved with a cover slip. Immunofluorescent images were obtained using Zeiss LSM700, as previously described [56].

Organoids were redispersed and resuspended into two-well chamber slides (Nunc Lab-Tek, Thermofisher, Waltham, MA, USA), allowed to reach 60–80% confluence, and then fixed with 4% paraformaldehyde (PFA) for 30 min. The PFA was then removed and the PDOs were washed 3 times with PBS, followed by incubation for 3 h in blocking solution. The blocking solution is PBS complemented with 1% (*v*/*v*) Triton-X-100, 1% (*w*/*v*) BSA, 1% (*v*/*v*) DMSO, and 1% (*v*/*v*) goat serum. Then, PDOs were stained overnight at 4 °C with primary antibodies against CDX2 (1:250, Dako IR080, Glostrup, Denmark) and CK20 (1:250, Ventana, SP33, Tucson, AZ, USA). After that, the primary antibody solutions were removed, and the chamber slides were washed 3 times with PBS/0.1% BSA. Secondary antibodies goat anti-mouse 488 (1:500, Invitrogen, AlexaFluor, #A11001, Waltham, MA, USA) and goat anti-rabbit 568 (1:500, LifeTechnologies, AlexaFluor, #A11036, Waltham, MA, USA) were added and the slides were incubated for 2 h at room temperature in the dark. Then, the secondary antibody solutions were removed, and the slides were washed 3 times with PBS/0.1% BSA. Finally, DAPI (1:2000) was applied for nuclear staining. Images were obtained using Zeiss LSM700, as previously described [56].

### 4.7. Reverse Transcriptase–Quantitative Polymerase Chain Reaction (RT-qPCR)

The confluent wells (6-well plate) of CRLM PDOs were treated with control medium (human liver expansion media) and treatment media (human liver expansion medium supplemented with 1 mM of captopril, 5 mM of captopril, or 10 mM of captopril) for 72 h following the same protocol as detailed above for the PDO viability assay. After 72 h, organoids were redispersed and resuspended in 1 mL of Trizol (Invitrogen, #15596-018) and stored at −80 °C. RT-qPCR was performed using the SYBR green PCR master mix and the cDNA was synthesized as previously described [56]. Briefly, the total RNA was isolated and DNAse treated using the Qiagen RNeasy Mini Kit (Qiagen, #74104, Hilden, Germany). The concentration of the total RNA was identified by a Nanodrop spectrophotometer. The cDNA was synthesized as follows. For each 1 μg of RNA, 1 μg of anchored primer mix was used. RNAse-free MilliQ water was used to make this up to a volume of 22 μL. The 22 μL mixture was heated at 70 °C for 5 min and then immediately placed on ice for 5 min. After that, 78 μL of reaction mix comprising 5X M-MLV buffer (Promega, #M531A, Madison, WI, USA), 10 mM of dNTP, RNAsin (40 U/μL) (Promega, #N2515, Madison, WI, USA), 1 M of DTT, and M-MLV reverse transcriptase enzyme (200 U/μL) (Promega, #M3682, Madison, WI, USA) in MilliQ water was added. The mixture was incubated at room temperature for 10 min, then at 42 °C in a heat block for 50 min to synthesize the cDNA. The gene expression levels were calculated relative to the housekeeping gene, *hydroxymethyl-bilane synthase* (*HMBS*) [57]. The expression levels are shown as fold *HMBS* expression and were calculated from the cycle threshold (Ct) values for *HMBS* (2^−ΔCT^) [56].

### 4.8. Statistical Analysis

GraphPad Prism was used for all statistical analysis and graphs. Data are expressed as the mean ± standard error of the mean (SEM) and were analyzed using the Student’s *t*-test. *p* values less than 0.05 were considered statistically significant.

## 5. Conclusions

This study demonstrates the establishment of CRLM PDOs which recapitulated parent tissue histopathology, and all responded to treatment with the RASi captopril. These findings corroborate our in vivo mouse experiments. The addition of a RASi to current treatment modalities for patients undergoing liver resection for CRLM should be considered.

## Figures and Tables

**Table 1:** Patient-derived organoids were generated from three patients undergoing liver resection for colorectal liver metastasis.

Patient	Age	Gender	CRC Stage *	Metachronous (M) or Synchronous (S) Lesion	Final Histopathology ^	MMR	KRAS	BRAF	Neoadjuvant Chemotherapy ^#^
#1	49	F	T3N1	M	Moderately differentiated adenocarcinoma, CRC metastasis	Proficient	WT	WT	No (adjuvant FOLFOX)
#2	75	F	T2N1	M	Moderately differentiated adenocarcinoma, CRC metastasis	Proficient	WT	^◊^	FOLFOX (6 cycles)
#3	58	M	T4N1	S	Moderately differentiated adenocarcinoma, CRC metastasis	Proficient	Mutant	WT	FOLFIRI (12 cycles)

* Primary colorectal cancer pathological staging. T—Tumor, N—nodes. ^^^ Final histopathology for liver resection specimens. ^#^ Chemotherapy administered prior to liver resection. ^◊^ BRAF testing not performed for this patient. MMR—DNA MisMatch Repair genes, proficient = no abnormalities in MMR genes detected. Patient demographics, final histology, and neo-adjuvant treatment are detailed here.

## Data Availability

Datasets will be made available by the corresponding author upon reasonable request.

## Supplementary Materials

The following supporting information can be downloaded at: https://www.mdpi.com/article/10.3390/ijms25063282/s1.
