# Supplementary material for: Renin–Angiotensin Inhibitor, Captopril, Attenuates Growth of Patient-Derived Colorectal Liver Metastasis Organoids
Source: Int J Mol Sci. 2024 Mar 14;25(6):3282. doi: 10.3390/ijms25063282 (PMC10970006; doi:10.3390/ijms25063282)
Supplement: Supplementary file 1 [file ijms-25-03282-s001.zip › ijms-2889688-supplementary.pdf]

International Journal of *Molecular Sciences*

MDPI

Supplementary Material

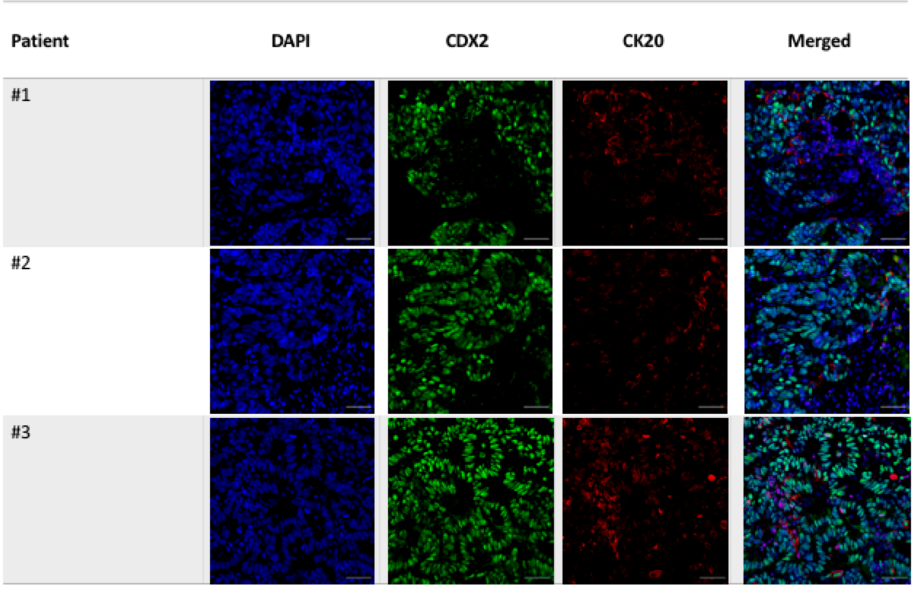

Figure S1. Parent tissue immunofluorescence. Patient-derived organoids were generated from 3 patients undergoing liver resection for colorectal liver metastasis. Part of the specimens obtained from each patient's tumour was formalin fixed and another part utilised for organoid generation. Sections of the formalin fixed parent tumours were obtained and used for immunofluorescence staining. Immunofluorescence staining with CDX2 and CK20 confirmed colorectal cancer origin and demonstrated cytoplasmic CDX2 staining and membranous CK20 staining. Scale bars = 50µm.

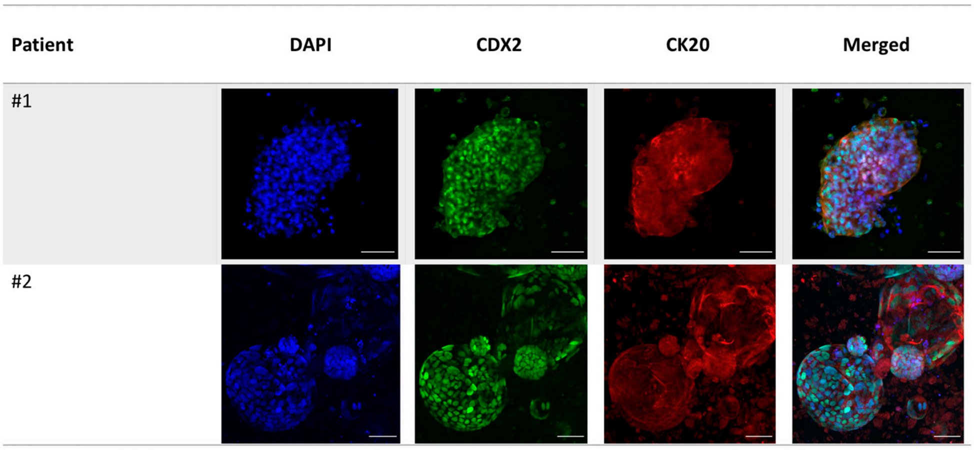

Figure S2. Patient-derived organoid immunofluorescence. Patient-derived organoids (PDOs) were generated from 3 patients undergoing liver resection for colorectal liver metastasis. For the first two patient-derived organoids immunofluorescence (IF) staining with CDX2 and CK20 confirmed colorectal cancer origin. IF staining of the PDOs followed the same pattern exhibited by the parent fixed tissue. Scale bars = 50µm.

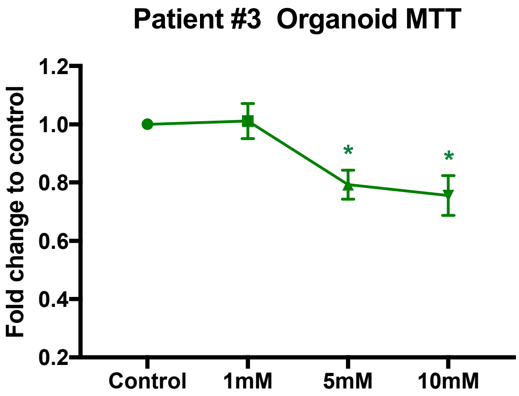

Figure S3. Patient #3 Organoids. Patient-derived organoids (PDOs) were established from the tumour of Patient #3 who underwent liver resection for colorectal liver metastasis (CRLM). Fresh tissue specimens were obtained and used to establish organoid cultures and a portion of parent tissue was formalin fixed. Patient #3 PDOs were treated with increasing concentrations of captopril and CRLM PDO growth assessed using the MTT assay. 3 biological repeats were performed (3 separate passages) and 8 technical replicates of each assessed. Statistical significance was determined using the Student's T test. *$p < 0.05$.

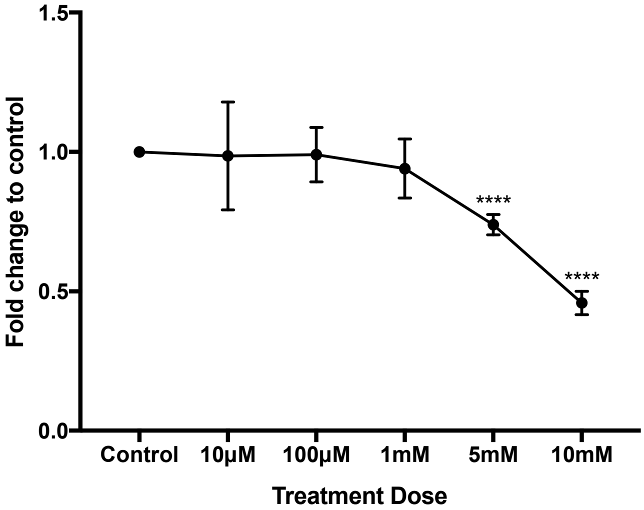

Figure S4. Viability assay characterisation was performed on patient-derived organoid #1. Patient-derived organoids were established from a patient undergoing liver resection for colorectal liver metastasis and used for organoid viability assays to establish which doses of captopril may attenuate organoid growth *in vitro*. Statistical analysis was performed using the Student's T test comparing each treatment to control, ****$p<0.0001$, n = 3 technical repeats.

	CONTROL	1MM CAPTOPRIL	10MM CAPTOPRIL
AXIN2	34.4	33.3	33.3
SOCS3	33.9	32.7	31.9

Supp. Table S1. RT-qPCR Patient #3. Raw data values shown as cycle threshold (Ct) values for genes *Axin2* and *Socs3*. The housekeeping gene *HMBS* was not detected due to mRNA/cDNA levels being below detection limits. High Ct values indicate weak gene expression detected late in the PCR cycle. Low Ct values indicate strong gene expression detected earlier in the PCR cycle.
